# Gut Microbial Signatures Can Discriminate Unipolar from Bipolar Depression

**DOI:** 10.1002/advs.201902862

**Published:** 2020-02-05

**Authors:** Peng Zheng, Jian Yang, Yifan Li, Jing Wu, Weiwei Liang, Bangmin Yin, Xunmin Tan, Yu Huang, Tingjia Chai, Hanping Zhang, Jiajia Duan, Jingjing Zhou, Zuoli Sun, Xu Chen, Subhi Marwari, Jianbo Lai, Tingting Huang, Yanli Du, Peifen Zhang, Seth W. Perry, Ma‐Li Wong, Julio Licinio, Shaohua Hu, Peng Xie, Gang Wang

**Affiliations:** ^1^ Department of Neurology The First Affiliated Hospital of Chongqing Medical University Chongqing 400016 China; ^2^ NHC Key Laboratory of Diagnosis and Treatment on Brain Functional Diseases Chongqing Medical University Chongqing 400016 China; ^3^ The National Clinical Research Center for Mental Disorders & Beijing Key Laboratory of Mental Disorders Beijing Anding Hospital Capital Medical University Beijing 100088 China; ^4^ Advanced Innovation Center for Human Brain Protection Capital Medical University Beijing 100088 China; ^5^ The M.O.E. Key Laboratory of Laboratory Medical Diagnostics the College of Laboratory Medicine Chongqing Medical University Chongqing 400016 China; ^6^ Department of Psychiatry and Behavioral Sciences College of Medicine State University of New York (SUNY) Upstate Medical University Syracuse NY 13210 USA; ^7^ Zhejiang University School of Medicine Hangzhou 310003 China; ^8^ Department of Psychiatry First Affiliated Hospital Zhejiang University School of Medicine Hangzhou 310003 China; ^9^ Department of Neuroscience & Physiology College of Medicine State University of New York (SUNY) Upstate Medical University Syracuse NY 13210 USA; ^10^ Department of Medicine College of Medicine State University of New York (SUNY) Upstate Medical University Syracuse NY 13210 USA; ^11^ Department Pharmacology College of Medicine State University of New York (SUNY) Upstate Medical University Syracuse NY 13210 USA; ^12^ The Key Laboratory of Mental Disorder's Management of Zhejiang Province Hangzhou 310003 China

**Keywords:** bipolar disorder, gut microbiome, major depressive disorder, microbiota–gut–brain axis, unipolar depression

## Abstract

Discriminating depressive episodes of bipolar disorder (BD) from major depressive disorder (MDD) is a major clinical challenge. Recently, gut microbiome alterations are implicated in these two mood disorders; however, little is known about the shared and distinct microbial characteristics in MDD versus BD. Here, using 16S ribosomal RNA (rRNA) gene sequencing, the microbial compositions of 165 subjects with MDD are compared with 217 BD, and 217 healthy controls (HCs). It is found that the microbial compositions are different between the three groups. Compared to HCs, MDD is characterized by altered covarying operational taxonomic units (OTUs) assigned to the Bacteroidaceae family, and BD shows disturbed covarying OTUs belonging to Lachnospiraceae, Prevotellaceae, and Ruminococcaceae families. Furthermore, a signature of 26 OTUs is identified that can distinguish patients with MDD from those with BD or HCs, with area under the curve (AUC) values ranging from 0.961 to 0.986 in discovery sets, and 0.702 to 0.741 in validation sets. Moreover, 4 of 26 microbial markers correlate with disease severity in MDD or BD. Together, distinct gut microbial compositions are identified in MDD compared to BD and HCs, and a novel marker panel is provided for distinguishing MDD from BD based on gut microbiome signatures.

## Graphical abstract

Gut microbiome alterations are implicated in major depressive disorder (MDD) and bipolar disorder (BD); however, little is known about the shared and distinct microbial characteristics in MDD versus BD. Here, the distinct microbial covarying units for MDD and BD are first identified, and microbial markers for distinguishing MDD from BD based on gut microbiome signatures are provided.

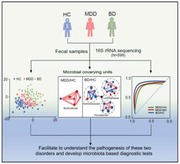

## 1 Introduction

Major depressive disorder (MDD) and bipolar disorder (BD) are two debilitating mood disorders for which correct diagnosis and distinct treatment regimes are critical, yet their overlapping symptomology makes rapid and accurate clinical diagnosis often difficult.^1^, ^2^, ^3^ The underlying molecular basis of the two mood disorders remains largely obscure. Moreover, diagnosis of MDD and BD currently relies on evaluation of symptoms and a clinical interview, which leads to high rates of misdiagnosis.^4^ Differential diagnosis of depressive episodes of BD and MDD is of great importance, since the pharmacotherapeutic strategies for relieving depressive symptoms in these two disorders are substantially different.^5^, ^6^ Antidepressants and mood stabilizers are the first choice for MDD and BD, respectively.^5^ Nonideal treatment resulting from initial misdiagnosis may aggravate the conditions, which, along with already considerable suicide or self‐harm risk in severe cases of MDD or BD, add further urgency to the need to “get it right the first time” rather than the current largely “trial and error” approach to initial pharmacotherapy. Therefore, identification of novel molecular signatures (biomarkers) for accurate differential diagnosis of MDD and BD is of enormous clinical importance.

The gastrointestinal (GI) tract is a complex ecosystem housing millions of resident microorganisms,^7^ collectively termed the “gut microbiome.” Compositional variations or other dysregulations of the gut microbiome are increasingly believed to play key roles in the pathogenesis of a growing number of enteric, metabolic, and psychiatric diseases.^8^, ^9^, ^10^, ^11^, ^12^, ^13^ Microbial biomarkers have been shown useful for identifying novel diagnostic and differential diagnostic tools, for these disorders.^10^ The gut microbiome broadly regulates healthy brain function and behavior,^14^, ^15^, ^16^ and has been implicated in the pathology of various neuropsychiatric disorders such as Parkinson's disease, stroke, autism, and schizophrenia via the microbiota–gut–brain (MGB) axis.^17^, ^18^, ^19^, ^20^, ^21^

Recent emerging evidence showed major shifts in the gut microbial composition of patients with MDD or BD, in small medication‐free cohorts.^22^, ^23^, ^24^, ^25^ For example, these studies showed that patients with MDD were characterized by enriched Enterobacteriaceae and *Alistipes*, and depleted *Faecalibacterium*, *Coprococcus*, and *Dialister*, relative to healthy control (HCs).^25^, ^26^ Furthermore, our previous studies have demonstrated that gut microbiome dysbiosis may contribute to depressive‐like behaviors.^22^, ^23^, ^27^ However, thus far, no studies have directly compared the microbial compositions of MDD and BD. Such investigations would be particularly valuable to understand the shared and distinct microbial characteristics between these two disorders, and to further identify microbial markers that might differentiate MDD, BD, and HC subjects.

Here, we performed a case–control study using 16S ribosomal RNA (rRNA) gene sequencing of stool samples from (*n* = 599) individuals with MDD (*n* = 165) and BD (*n* = 217) compared with HCs (*n* = 217). Using linear discriminant analysis (LEfse), we sought to identify MDD‐ and BD‐related microbial signatures compared to HCs. Next, co‐occurrence analysis based on the relative abundance of altered bacterial operational taxonomic units (OTUs) was performed to construct the key covarying networks in MDD and BD. Finally, we sought to identify a discriminative microbial panel that could distinguish MDD from BD and HCs, then further validate the diagnostic performance of this gut microbiome signature using discovery and validation set samples, respectively.

## 2 Results

### 2.1 Clinical Characteristics of Recruited Participants

A total of 599 subjects were recruited for this study (Table S1, Supporting Information). In the discovery set (*N* = 462), MDD, BD, and HC subjects were matched for key demographic variables including age, gender, and body mass index (BMI), and all MDD and BD subjects were unmedicated and experiencing depressive episodes, allowing us to isolate and identify the microbial signatures that reflect only the pathophysiologic variations inherent in MDD and BD, without influence of confounding variables. In the validation set (*N* = 137), demographic variables including age, gender, and BMI were purposefully not controlled for, and some patients with MDD and BD were medicated (but still experiencing a depressive episode). This “uncontrolled” validation set allows us to assess and confirm the diagnostic performance of an identified microbial signature under “real world” conditions. The overall workflow is shown in Figure S1 (Supporting Information).

### 2.2 Comparison of α‐Diversity among MDD, BD, and HC

**Figure 1:**
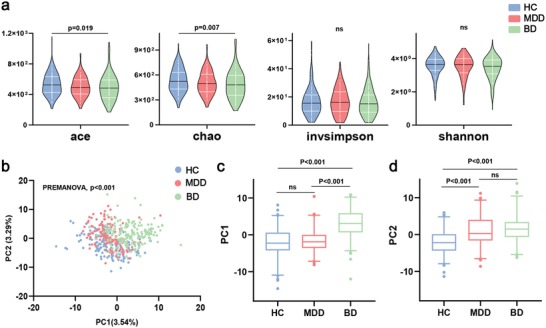
Differential gut microbial characteristics in major depressive disorder (MDD; *n* = 122), bipolar disorder (BD; *n* = 169), and healthy controls (HCs; *n* = 171). a) Violin plots of α‐phylogenetic diversity showed that two indices (Ace and Chao) were decreased in BD subjects relative to HCs, suggesting a lower α‐diversity in BD. There were no significant differences in α‐diversity indices between MDD and BD (multiple comparisons, one‐way ANOVA). b) PLS‐DA analysis showed a clear separation between the three groups at the operational taxonomic units (OTU) level (PERMANOVA, *p* < 0.001). c) In the PC1 PLS‐DA analysis, the BD group was significantly different from MDD and HC groups. d) In the PC2 PLS‐DA analysis, both MDD and BD were significantly different from HC (multiple comparisons, Kruskal–Wallis test). Abbreviation: PC, Principal Component.

In the discovery set, we obtained 24 876 670 high‐quality reads across all samples, with an average length of 433.78. These reads were clustered into 3012 OTUs at 97% sequence similarity. The α‐diversity values including species richness indices (Ace and Chao) and species diversity indices (Shannon and invsimpson) were compared among the MDD, BD, and HC groups. We found that the indices of Ace and Chao were decreased in patients with BD relative to HCs (*p* < 0.019 and *p* < 0.007, respectively; **Figure** **1**a). These indices were not different between the MDD and BD groups, or the MDD and HC groups (Figure 1a).

To explore whether the comprehensive microbial phenotypes of MDD, BD, and HC were different, β‐diversity analysis was performed. Partial least‐squares discriminant analysis (PLS‐DA) showed that the three groups could be distinguished at the OTU level (Figure 1b; permutational multivariate analysis of variance (PERMANOVA), *p* < 0.001). At the Principal Component 1 (PC1) PLS‐DA analysis, BD was significantly discriminated from both MDD and HC (*p* < 0.001, both; Kruskal–Wallis test) (Figure 1c). At the PC2 PLS‐DA analysis, both MDD and BD were significantly different from HC (*p* < 0.001, both; Kruskal–Wallis test) (Figure 1d). Control analyses showed that the overall microbial phenotypes of the MDD, BD, and HC groups were not significantly influenced by potential confounders such as body mass index, recruitment sites, or age (Figures S2 and S3a,b, Supporting Information). Moreover, samples from the discovery sets were well intermixed with samples from the corresponding validation sets (Figure S3c–e, Supporting Information), thus ruling out potential biases caused by set classifications. Together these findings suggest that potential confounding variables did not greatly influence the overall microbiome compositions in our data.

### 2.3 Distinct Gut Microbiome Signatures in MDD and BD

**Figure 2:**
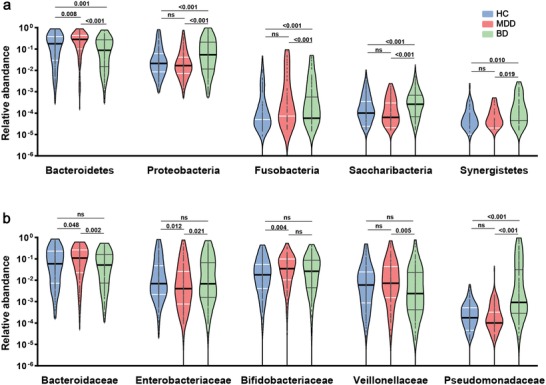
Altered levels of specific bacterial phyla and families in MDD, BD, and HC. a) Two dominant bacterial phyla including Bacteroidetes and Proteobacteria were altered among the three groups. Bacteroidetes were significantly decreased in BD versus MDD or HC. In contrast, Proteobacteria was upregulated in BD relative to MDD or HC. Bacteroidetes but not Proteobacteria levels were different between HC and MDD. Additionally, three less abundant phyla including Saccharibacteria, Fusobacteria, and Synergistetes were all different between MDD and BD, and BD and HC, but not MDD and HC. b) Five representative most abundant family‐level phylotypes across the three groups were shown. Bacteroidaceae and Bifidobacteriaceae were higher, and Enterobacteriaceae lower, in MDD versus HC. Only Pseudomonadaceae was different (higher) in BD versus HC. Bacteroidaceae and Veillonellaceae were higher, and Enterobacteriaceae and Pseudomonadaceae lower, in MDD versus BD. (All multiple comparisons, Kruskal–Wallis test.).

Next, the relative abundance of microbial compositions was compared among the three groups in the discovery set at both the phylum and family levels (**Figure** **2**). We found that, at the phylum level (Figure 2a), Bacteroidetes were significantly depleted in the BD group compared with the MDD or HC groups. In contrast, Bacteroidetes were higher in MDD relative to HC. The relative abundance of Proteobacteria was enriched in the BD group relative to the MDD or HC groups. There was no difference in Proteobacteria between the HC and MDD groups. Additionally, three less abundant phyla including Saccharibacteria, Fusobacteria, and Synergistetes were also changed to different degrees. At the family levels, we identified 37 families to be differentially abundant in the pairwise comparisons between the three groups (Table S2, Supporting Information). Five representative high‐abundance families are shown in Figure 2b. For BD, only Pseudomonadaceae levels were significantly different (higher) than HC. For MDD, Bacteroidaceae and Bifidobacteriaceae were higher than HC, whereas Enterobacteriaceae was lower than HC. Comparing MDD and BD against each other, MDD had higher Bacteroidaceae and Veillonellaceae versus BD, whereas BD had higher Enterobacteriaceae and Pseudomonadaceae versus MDD.

**Figure 3:**
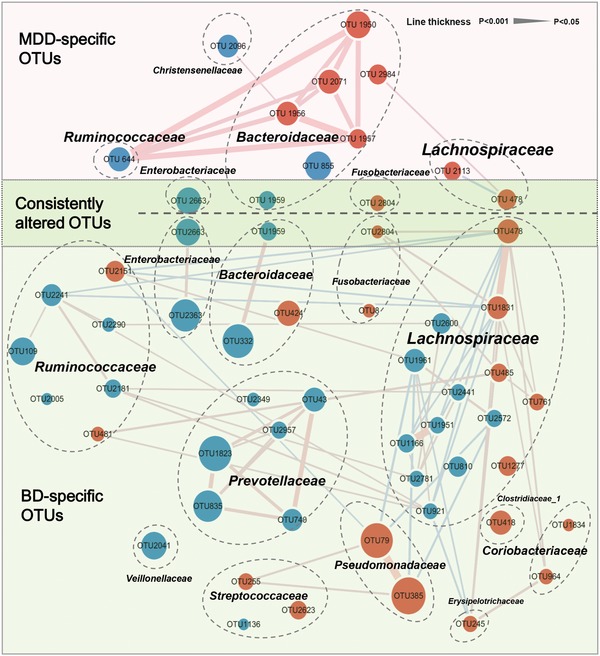
The co‐occurrence network reflecting microbial changes in MDD, BD, and HC. The microbial OTUs changed in MDD or BD were identified by LDA (LDA > 2.5; fold change > 2). In total, 57 differential OTUs were identified in the two groups. Four of 57 OTUs were consistently altered in both MDD and BD relative to HC (dark green area), while the majority of OTUs were specific to MDD alone (9/13) (pink area) or BD (40/44) alone (light green area). Compared to HC, MDD was mainly characterized by altered covarying OTUs assigned to the family Bacteroidaceae, while BD was mainly characterized by altered covarying OTUs assigned to the families Lachnospiraceae, Prevotellaceae, and Ruminococcaceae. Size of the nodes scales with the relative abundance of the OTU. Red dots: increased relative abundance in MDD or BD compared to HC; blue dots: decreased relative abundance in MDD or BD relative to HC. OTUs annotated to family level were marked. Lines between nodes indicate Spearman's correlation < −0.35 (light blue), or > +0.35 (light red); line thickness indicates *p* value (*p* < 0.05).

To characterize the shared and distinct microbial compositions between MDD and BD in detail, we further identified key discriminative OTUs in MDD or BD subjects relative to HCs using LEfSe analysis. In total, we identified 57 OTUs to be differentially abundant in the MDD and BD groups compared to the HC group (Figure S4a,b and Tables S3 and S4, Supporting Information). Among them, only four OTUs (Enterobacteriaceae_OTU2663, Lachnospiraceae_OTU478, Bacteroidaceae_ OTU1959, and Fusobacteriaceae _OTU2804) were consistently changed in both MDD and BD (**Figure** **3**). By contrast, the majority of altered OTUs were specific to patients with either MDD (9/13) or BD (40/44). Compared to HC, MDD was characterized by enriched OTUs belonging to the families Bacteroidaceae (five OTUs) and Lachnospiraceae (two OTUs), and depleted OTUs belonging to the family Bacteroidaceae (two OTUs) (Table S3, Supporting Information). BD was characterized by increased OTUs belonging to the families Lachnospiraceae (five OTUs), Streptococcaceae (two OTUs), and Ruminococcaceae (two OTUs), and decreased OTUs belonging to the families Lachnospiraceae (nine OTUs), Prevotellaceae (six OTUs), Ruminococcaceae (five OTUs), and Bacteroidaceae (two OTUs) (Table S4, Supporting Information).

Here, co‐occurrence network analysis provides an illustration of the statistical covariation among altered OTUs (Figure 3). In the MDD group, we found that five Bacteroidaceae OTUs (OTU1950, 1956, 1957, 2071, and 2984) and one Ruminococcaceae OTU (OTU 644) positively covaried with one another, which generated a characteristic covarying network mostly from Bacteroidaceae OTUs. In the BD group, the covarying networks constructed by altered OTUs were relatively complex and diverse. The BD group displayed disturbed covarying OTUs mainly belonging to families Lachnospiraceae, Prevotellaceae, and Ruminococcaceae. Interestingly, we found that the six Prevotellaceae OTUs (OTU43, 740, 835, 1823, 2349, and 2957) were consistently decreased in BD relative to HC. The majority of Prevotellaceae OTUs (5/6) were also positively correlated with one another. Moreover, the covarying networks generated by Lachnospiraceae and Ruminococcaceae OTUs were robust but complex, as the OTUs belonging to the two families were both up‐ and downregulated in BD relative to HC, and also both positively and negatively correlated with one other.

To confirm the microbial differences between the MDD and BD groups, direct comparison between the two groups was performed. Here, MDD showed 19 increased OTUs and 15 decreased OTUs relative to BD (Figure S4c and Table S5, Supporting Information). These 34 differential OTUs clustered mainly into two covarying networks comprised of eight Bacteroidaceae OTUs and eight Lachnospiraceae OTUs (Figure S5, Supporting Information). Together, these results indicate that MDD and BD share a small proportion of their gut microbial phenotypes, but have significantly different microbial signatures.

### 2.4 Gut Microbial Biomarkers for Discriminating MDD, BD, and HC

**Figure 4:**
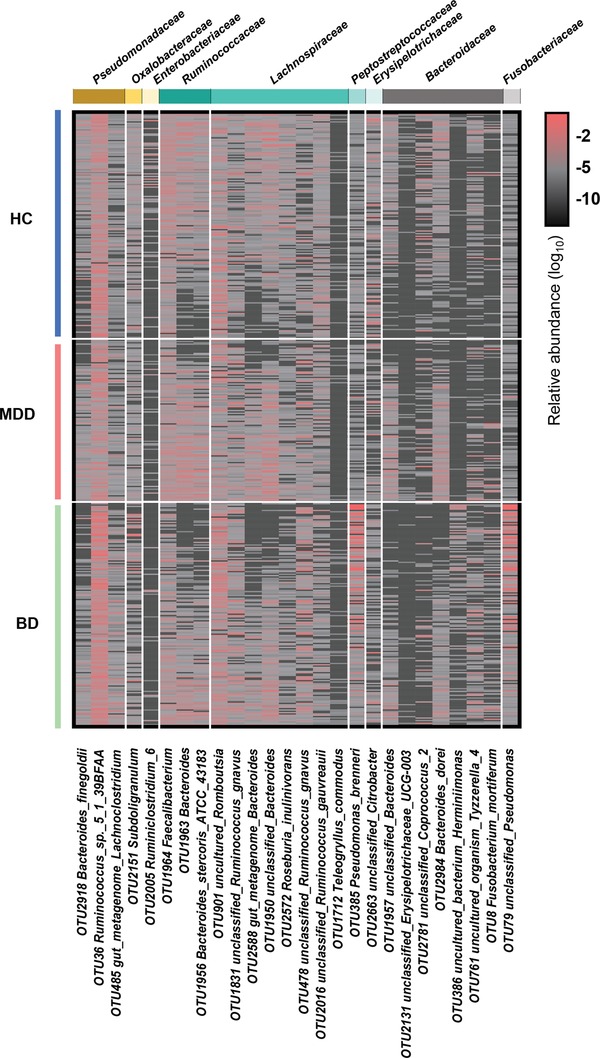
Differential diagnostic microbial markers for discriminating MDD, BD, and HC. Using the LEfSe analysis with LDA score > 2.5, 26 discriminative OTUs for MDD, BD, and HC were identified and designated as the candidate diagnostic markers. These discriminative OTUs belonged mainly to the families Lachnospiraceae (eight OTUs), Bacteroidaceae (seven OTUs), Pseudomonadaceae (three OTUs), and Ruminococcaceae (three OTUs).

**Figure 5:**
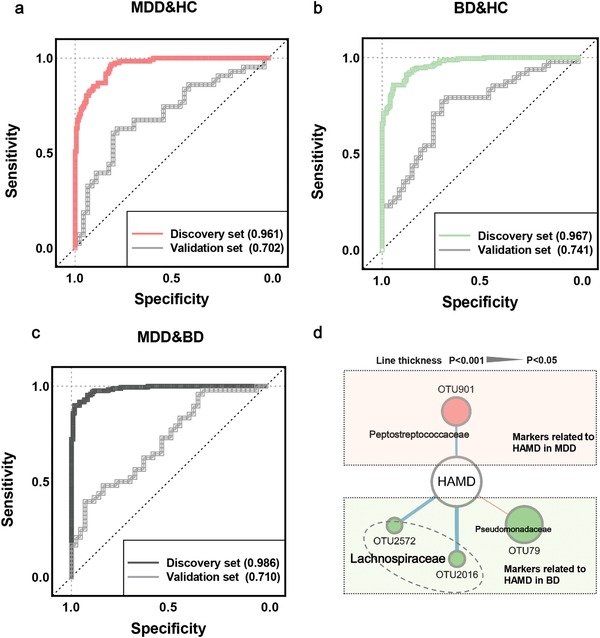
Discovery and validation set AUCs reflect the differential diagnostic potential of microbial markers for discriminating MDD, BD, and HC. a–c) Random Forest analysis was used to quantify the diagnostic performance. In the discovery set, this microbial panel enabled distinguishing MDD from BD or HC with high diagnostic accuracy (MDD vs HC, AUC = 0.961; BD vs HC, AUC = 0.967; MDD vs BD, AUC = 0.986). (Discovery set: HC, *n* = 171; MDD, *n* = 122; BD, *n* = 169). The diagnostic performance of this microbial classifier was further tested in a validation set. The validation set confirmed diagnostic performance with AUC values of at least 0.7 (MDD vs HC, AUC = 0.702; BD vs HC, AUC = 0.741; MDD vs BD, AUC = 0.710). (Validation set: HC, *n* = 46; MDD, *n* = 43; BD, *n* = 48). d) Four microbial OTUs, mainly belonging to the family Lachnospiraceae, were significantly associated with HAMD in MDD or BD patients. Red lines indicate positive associations between these microbial OTUs and clinical indices, blue lines indicate negative associations. The statistical significance was denoted on the width of lines (*p* < 0.05). Abbreviation: HAMD, Hamilton Depression Rating Scale.

To identify microbial signatures able to discriminate MDD and BD from each other, as well as from HCs, the differential OTUs among the three groups were analyzed using LEfSe analysis (Figure S4d and Table S6, Supporting Information). In total, we identified 26 differentially abundant OTUs among the three groups. These discriminative OTUs belonged mainly to the Lachnospiraceae (eight OTUs), Bacteroidaceae (seven OTUs), Pseudomonadaceae (three OTUs), and Ruminococcaceae (three OTUs) families (**Figure** **4**). Next, these 26 differential OTUs were analyzed by random forest classifier to quantify their diagnostic performance using the area under the curve (AUC). In the discovery set (all unmedicated and with matched controls), this microbial panel enabled distinguishing the subjects with MDD from those with BD or HC with very high diagnostic accuracy (MDD vs HC, AUC = 0.961; BD vs HC, AUC = 0.967; MDD vs BD, AUC = 0.986; **Figure** **5**a–c). Internal validation using random subsets of this discovery set confirmed the discriminative ability of this marker panel (Figure S6, Supporting Information). Next, the diagnostic efficiency of this microbial classifier was tested in a validation set (external validation). In this “real world” validation set, we found that this microbial marker panel could still effectively differentiate the MDD, BD, and HC groups with AUCs ranging from 0.702 to 0.741 (MDD vs HC, AUC = 0.702; BD vs HC, AUC = 0.741; MDD vs BD, AUC = 0.710; Figure 5a–c).

To further determine whether the 26 discriminative markers could reflect disease severity, Spearman's rank correlation analysis was performed. We found that four microbial OTUs, mainly belonging to the family Lachnospiraceae, were significantly associated with Hamilton Depression Rating Scale (HAMD) in MDD or BD patients (Figure 5d). Together, we identified the microbial markers that enabled discriminating the MDD, BD, and HC groups, and some microbial markers reflected the severity of patients with MDD or BD.

## 3 Discussion

In this study, using well‐characterized and large cohorts, we, for the first time, characterized the gut microbial composition of MDD and BD compared to each other and HC. We identified unique microbial signatures of subjects with MDD and BD relative to HC. Moreover, we identified and confirmed a gut microbial classifier with diagnostic and differential diagnostic potential, which can distinguish MDD from BD, and each from HC, with high accuracy. This result is worth further exploration in clinical trials. Aside from this exciting potential for clinical diagnostic utility, these results may also help identify novel therapeutic targets for MDD and BD.

Changes in gut microbiome composition have been demonstrated in various diseases, including MDD and BD, but we believe this is the first large study to compare gut microbiome composition in MDD versus BD, and each versus HC. Herein, we found that the indices of α‐diversity (Ace and Chao) were decreased in BD relative to HC. Supporting this finding, lower gut microbial indices of α‐diversity (Chao and Obs) were also observed in a previous smaller sample size study of just BD versus HC.^23^ Moreover, both this current and our previous^22^ studies found no α‐diversity differences between MDD and HC. In our previous study, MDD was characterized by alterations in specific OTUs assigned to the phyla Firmicutes, Actinobacteria, and Bacteroidetes.^22^ Likewise, here we found that MDD was associated with disturbances of Bacteroidetes and Firmicutes (Table S3, Supporting Information). Further, at the family level, increased Lachnospiraceae and decreased Bacteroidaceae OTUs were consistently observed in both this and our previous report,^22^ but five increased Bacteroidaceae OTUs were newly correlated with MDD in this current study.

In terms of BD, previous studies found that BD was mainly associated with disturbances of Firmicutes and Bacteroidetes phyla.^23^ Consistent with this finding, we found that Firmicutes, Bacteroidetes, and Proteobacteria phyla were altered in BD relative to HC. Moreover, at the genera levels, *Bacteroides*, *Roseburia*, and *Coprococcus* were consistently changed in BD relative to HC. Here we also newly report genera such as *Prevotella*_9 and *Pseudomonas* as associated with BD (Table S4, Supporting Information).

As is often the case with investigations of disease metagenomics,^10^ a few inconsistencies with previous reports can be identified. For example, some genera such as *Parabacteroides*, previously identified as higher in MDD versus HC,^28^ were not observed here. Overall, however, our findings here are consistent with the microbial signatures of MDD and BD as found in previous studies, despite cross‐study differences in inclusion criteria, sequencing platforms, and cohort demographics. Furthermore, here we newly report some previously unidentified microbial signatures associated with MDD or BD. This current work lays a foundation from which to further characterize the shared and distinct microbial underpinnings of MDD and BD.

Besides identifying the microbial compositions that characterized each disease, we wanted to know what microbial biomarkers could discriminate MDD and BD (from each other and from HC), for further development as a potential diagnostic tool. Toward this end, we identified a signature of 26 OTUs that could distinguish patients with MDD from those with BD or HCs, with AUC values ranging from 0.961 to 0.986 in discovery sets, and 0.702 to 0.741 in validation sets (with exact AUC value depending on the comparison made). We believe this method has potential as a first‐of‐its‐kind rapid, noninvasive diagnostic and differential diagnostic tool for distinguishing MDD from BD (and both from HC) based on subjects' gut microbiome composition, which would fill a significant and currently unmet clinical need for better quantitative diagnostic tools to quickly distinguish MDD from BD in order to optimize the initial treatment approach.

Our data also provide mechanistic insights. Compared with the HC group, the MDD group had differential covarying OTUs assigned to family Bacteroidaceae, while BD showed altered covarying OTUs belonged to families Lachnospiraceae, Prevotellaceae, and Ruminococcaceae. In the MDD group, there was a positive correlation network among the five upregulated Bacteroidaceae OTUs and one downregulated Enterobacteriaceae OTU. This finding suggests that these OTUs may play a cooperative role in the gut microbial environment of MDD. Moreover, disrupted Bacteroidaceae OTUs were a hallmark in the gut ecosystem of MDD. Bacteroidaceae can induce cytokine production, and enriched Bacteroidaceae OTUs may be linked with higher peripheral cytokine levels and increased inflammation in MDD.^29^ In the BD group, the correlation networks formed by altered OTUs were relatively complex and diverse, with the major downregulated Prevotellaceae OTUs positively correlated with one another. These results suggest that further studies should focus on both the function of disease‐specific strains belonging to Bacteroidaceae and Prevotellaceae, but also how these strains regulate the entire microbial networks, especially different strains under the same microbial taxonomy.^30^ This, together with our results here, may uncover novel therapeutic targets for BD and MDD.

To identify the microbial biomarkers that most accurately reflect the underlying pathology of MDD and BD, and because emerging studies have shown that gut microbial composition may be influenced by antidepressants and antipsychotics,^26^, ^31^, ^32^ only well‐matched unmedicated subjects were recruited in the discovery set. In the validation set, to better confirm the diagnostic generalizability of our differential diagnostic assay in a closer‐to‐real‐world scenario, the samples were not matched on age and gender. This dual strategy is valuable for fully evaluating the diagnostic performance of microbial markers. Here, we found that the AUC values in the discovery set were higher than in the validation set. This finding is expected without control matching, and highlights that this kind of independent validation is both necessary and useful to avoid prevailing issues of overly optimistic reports of diagnostic accuracy. In this study, the microbial marker panel derived from the discovery set maintained its accuracy in discriminating samples from the validation set, suggesting that this microbiota‐based diagnostics assay might be generalizable in clinical application.

We acknowledge the following strengths and limitations of our study. A major strength of this study is that the sample size of unmedicated MDD and BD subjects was large, and the recruited subjects in the training set were strictly control‐matched with well‐characterized clinical information. These measures help eliminate bias arising from potential confounding effects such as medication status, age, and BMI. Moreover, non‐control‐matched subjects were used to independently verify the diagnostic efficiency of microbial markers in the validation set, which is the premise of multicenter validation of candidate microbial classifiers and another strength of this work. Because one's individual (and collective) environmental variables such as geography and diet may influence their gut microbiome composition, we recruited some subjects from two geographically distinct sites and found their overall microbiome did not cluster significantly by region/site, another strength. Potential limitations of this study: i) environmental or site‐specific biases on subjects' microbial compositions cannot be completely ruled out; ii) similarly, due to lack of detailed dietary information for the subjects, our study could not control for or assess whether and how dietary habits influence gut microbial composition in MDD, BD, or HC; iii) our findings cannot (and did not endeavor to) show a causal relationship between the identified differential gut microbial compositions and BD or MDD, a limitation that is inherent to any cross‐sectional studies of this nature. Useful future studies to extend and advance this current work might include a longitudinal study to assess how microbial composition varies with clinical improvement in medicated MDD and BD subjects; and iv) based on this well‐characterized and larger sample cohort, some emerging analytical strategies should be considered.

## 4 Conclusion

In summary, herein we have characterized and identified different gut microbial compositions in subjects with MDD versus BD, and in both versus HC. Moreover, we have developed and independently validated a gut microbial classifier able to effectively discriminate MDD from BD and HC. Our findings lay the foundation of further development of a much‐needed gut microbiota‐based clinical diagnostic assay for diagnosis and differential diagnosis of MDD and BD.

## 5 Experimental Section

##### Subject Recruitment

The protocols of this study were reviewed and approved by the Human Research and Ethics Committee of Beijing Anding Hospital of Capital Medical University (#2017‐24) and the First Affiliated Hospital, School of Medicine of Zhejiang University (#2017‐397). Each participant signed an informed consent, and the fecal samples were collected in the two research centers. Diagnosis of MDD and BD was performed based on Structured Clinical Interview for DSM‐IV criteria by two senior psychiatrists as in the previous studies.^23^, ^33^ Here, all the patients with MDD were undergone depressive episodes (abbreviated as MDD). The BD patients with a current depressive but not manic episode were recruited (abbreviated as BD), and classified into two subtypes (BD‐I and BD‐II). The HAMD and Young Manic Rating Scale (YMRS) were used to evaluate the severity of MDD or BD.^34^, ^35^ The HCs were recruited from advertising in the two centers. All participants did not have any physical or other mental disorders or illicit drug use, and they also had not taken antibiotics, probiotics, or prebiotics within 1 month prior to sampling. In the discovery set, all patients with MDD and BD were unmedicated. By contrast, in the validation set, some MDD and BD patients were medicated, for reasons justified above. The detailed characteristics of these recruited subjects are shown in Table S1 (Supporting Information).

##### DNA Extraction, Polymerase Chain Reaction (PCR) Amplification, and Illumina MiSeq Sequencing

The Illumina MiSeq sequencing protocol was similar to the previously published studies.^34^, ^36^ Briefly, frozen fecal samples were used to extract the microbial DNA OMEGA‐soil DNA Kit (Omega Bio‐Tek, USA) according to manufacturer's protocols. The NanoDrop 2000 UV–vis spectrophotometer was used to determine the DNA concentration and purification, and DNA quality was checked by 1% agarose gel electrophoresis. The V3–V4 hypervariable regions of the bacteria 16S rRNA gene were amplified by PCR with the use of primers 338F (5′‐ACTCCTACGGGAGGCAGCAG‐3′) and 806R (5′‐GGACTACHVGGGTWTCTAAT‐3).^37^ PCR reactions were carried out in triplicate 20 µL mixtures. Primers included an eight base sequence unique to each sample. Amplicons were extracted from 2% agarose gels and purified using the AxyPrep DNA Gel Extraction Kit (Axygen Biosciences, Union City, CA, USA). Purified amplicons were quantified using QuantiFluor‐ST (Promega, USA) and paired‐end sequenced (2 × 250) on an Illumina MiSeq platform using standard protocols in Shanghai Majorbio Bio‐pharm Technology Co., Ltd.

##### 16S rRNA Gene Sequence Analysis

Raw fastq files were quality‐filtered by Trimmomatic and merged by fast length adjustment of short reads with the following standards: i) the reads were truncated at any site with an average quality score of <20 over a 50 bp sliding window; ii) sequences with overlaps of >10 bp were merged according to their overlap with mismatch no more than 2 bp; iii) sequences of each sample were separated based on barcodes and primers, and reads containing ambiguous bases were removed. The remaining high‐quality sequences were clustered into OTUs at 97% similarity using UPARSE (version 7.1 https://drive5.com/uparse/).^38^ The taxonomy of each 16S rRNA gene sequence was analyzed by ribosomal database project classifier algorithm (https://rdp.cme.msu.edu/). α‐diversity was assessed using the species richness indices (Ace and Chao) and species diversity indices (Shannon).^39^ PLS‐DA was used to explore the differences and similarities of microbial compositions among the three groups.^40^ To test statistical significance, the Kruskal–Wallis test was used for the two Principal Components obtained from the PLS‐DA model. The PERMANOVA test was used to test the group differences. LEfse was carried out to identify the different bacterial taxa among the three experimental groups.^41^

##### Microbial Markers for MDD and BD

The overall workflow of this study is shown in Figure S1 (Supporting Information). Initially, LEfse was used to identify the MDD‐ or BD‐related OTUs relative to HCs (linear discriminant analysis (LDA) > 2.5, fold change > 2). Then, the co‐occurrence networks deduced from the relative abundance of MDD‐ or BD‐related OTUs were generated using Spearman's correlation coefficient (*r* > 0.35 or <−0.35; *p* < 0.05) and visualized in Cytoscape V.3.7.0. Based on the resulting co‐occurrence network, not only MDD or BD specific networks could be identified, but also how these microbes in a particular network correlate with each other could be uncovered.

To identify discriminative microbial markers across the MDD, BD, and HC groups, the different OTUs among the three groups were analyzed using LEfse analysis. Then, all discriminative OTUs were input for the random forest classifier (Python's scikit‐learn package) to predict the discrimination between MDD/HCs, BD/ HCs, or MDD/BD. In each case, 1000 trees were considered (other scikit‐learn defaults were left unchanged). The receiver operating characteristic (ROC) curve was obtained (SPSS V.19.0) for the display of the constructed models, then the area under the ROC curve (AUC) was used to designate the ROC effect.^42^ Internal validation was performed as follows. The samples from the discovery set were divided into two subgroups which contained 80% and 20% of the subjects, respectively. In addition, external verification was also performed, as use of independent samples from the validation set is a necessary step prior to multicenter clinical validation.

##### Statistics

Statistical analyses were performed using SPSS version 21 (SPSS, Chicago, IL, USA). One‐way ANOVA was used to compare the continuous variables including age, BMI, and clinical scales. Categorical variable (gender) was analyzed by the χ^2^ test. The Kruskal–Wallis test was used to compare the relative abundances of microbes obtained from 16S rRNA sequence. PERMANOVA was performed on R studio (3.7.1) and package “vegan,” permutations = 9999, distance = “bray.” Outcomes were presented as mean ± standard deviation (SD). Statistical significance level was set at *p* < 0.05.

## Conflict of Interest

The authors declare no conflict of interest.

## Author Contributions

P.Z. and J.Y. contributed equally to this study. Designed the experiments: P.X., G.W., and S.H.H. Performed the 16S rRNA gene analysis: P.Z., Y.F.L., J.Y., W.W.L., B.M.Y., X.M.T., Y.H., T.J.C., H.P.Z., and J.J.D. Collected the clinical samples: J.Y., J.J.Z., Z.L.S., X.C., G.W., S.H.H., J.L., T.T.H., Y.L.D., and P.F.Z. Drafted the manuscript: P.X., P.Z., and J.Y. Revised the manuscript for intellectual content: S.M., S.W.P., J.L., and M.‐L.W.

## Supporting information

Supporting Information
