# Supplementary material for: Gut Microbial Signatures Can Discriminate Unipolar from Bipolar Depression
Source: Adv Sci (Weinh). 2020 Feb 5;7(7):1902862. doi: 10.1002/advs.201902862 (PMC7140990; doi:10.1002/advs.201902862)
Supplement: Supplementary file 1 — Supporting Information [file ADVS-7-1902862-s001.pdf]

Copyright WILEY-VCH Verlag GmbH & Co. KGaA, 69469 Weinheim, Germany, 2020.

ADVANCED SCIENCE
Open Access

Supporting Information

for *Adv. Sci.,* DOI: 10.1002/advs.201902862

Gut Microbial Signatures Can Discriminate Unipolar from Bipolar Depression

Peng Zheng, Jian Yang, Yifan Li, Jing Wu, Weiwei Liang, Bangmin Yin, Xunmin Tan, Yu Huang, Tingjia Chai, Hanping Zhang, Jiajia Duan, Jingjing Zhou, Zuoli Sun, Xu Chen, Subhi Marwari, Jianbo Lai, Tingting Huang, Yanli Du, Peifen Zhang, Seth W. Perry, Ma-Li Wong, Julio Licinio, Shaohua Hu, Peng Xie,* and Gang Wang**

Supplementary Materials

Supplementary Figures

Figure. S1. The workflow diagram for this work.
Figure. S2. The influence of potential confounders on overall microbial composition.
Figure. S3. The influence of potential confounder on overall microbial composition.
Figure. S4. Discriminative OTUs observed in the pairwise comparisons between the three groups.
Figure. S5. Co-occurrence networks deduced from the relative abundance of differential OTUs between MDD and BD.
Figure. S6. Validation of diagnostic performance of microbial markers in discovery set.

[Figures below]

Figure. S1

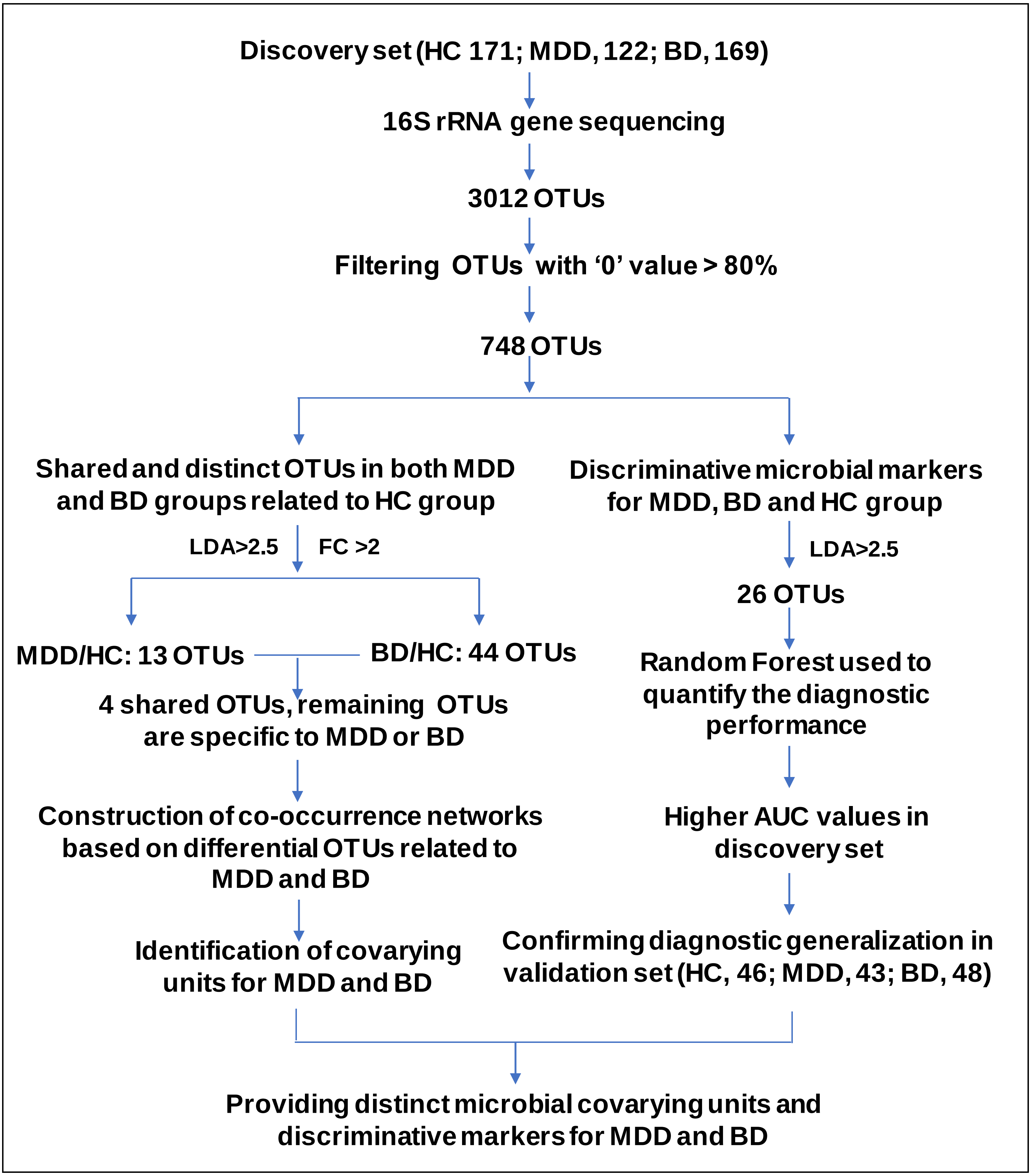

Figure. S1. The workflow diagram for this work. Abbreviation: HC, healthy controls; MDD, major depressive disorder; BD, bipolar disorder; OTU, operational taxonomic units; AUC, area under the curve; LEfSe, linear discriminant analysis effect size; FC, fold changes.

Figure. S2.

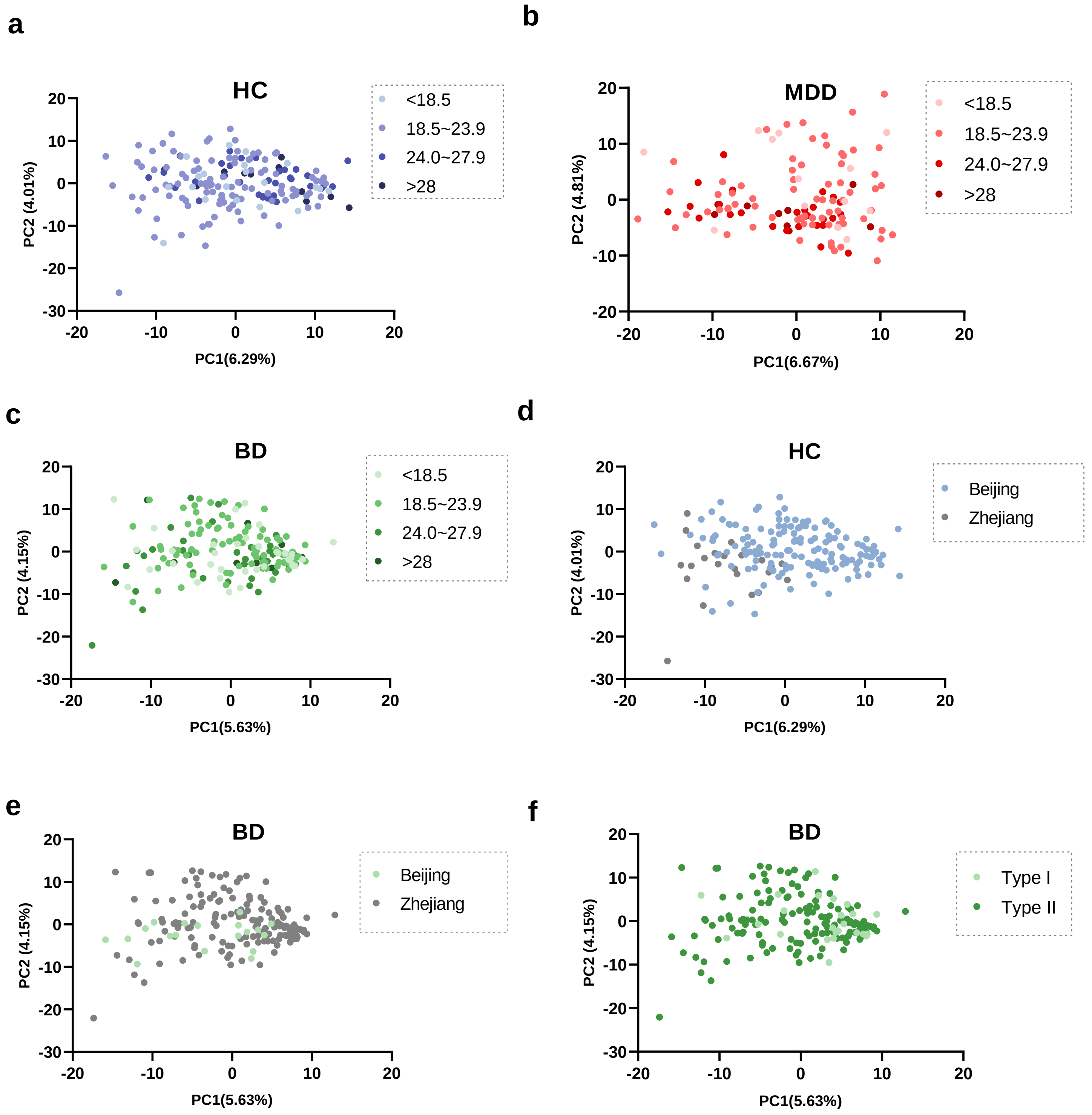

Figure. S2. The influence of potential confounders on overall microbial composition. (a-c) The samples from MDD, BD and HC groups were not clustered based on Body Mass Index. (d-e) The samples from BD and HC groups were not clustered based on research centers (Beijing or Zhejiang). (f) The samples of BD-I were almost mixed with samples of BD-II. Here, all samples were obtained from discovery set.

Figure. S3.

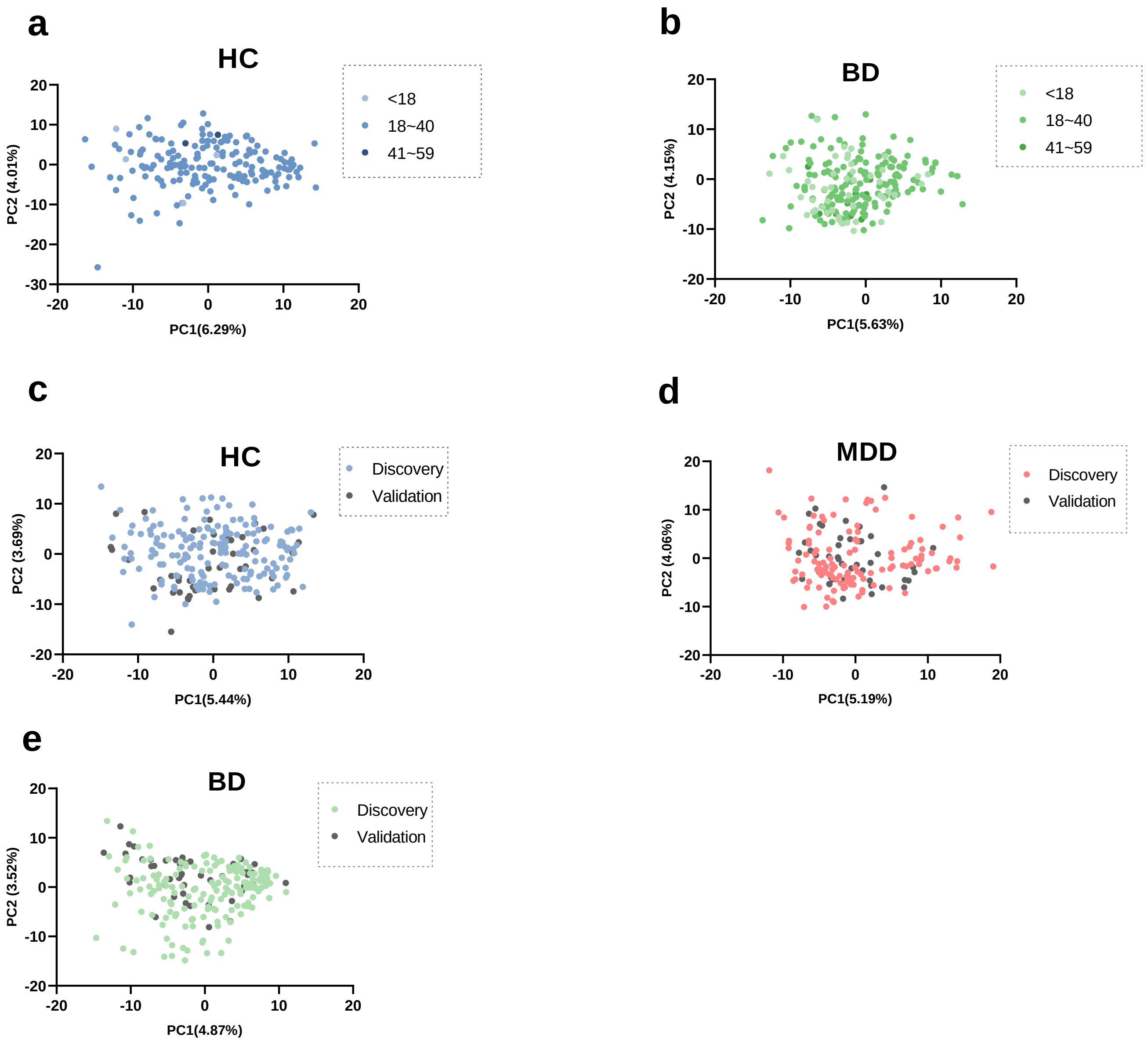

Figure. S3. The influence of potential confounders on overall microbial composition. (a-b)The samples from different age periods were mixed with the remaining samples in the respective group. (c-e) The samples in the validation set (grey dots) are mixed with the corresponding samples in the discovery set.

Figure. S4.

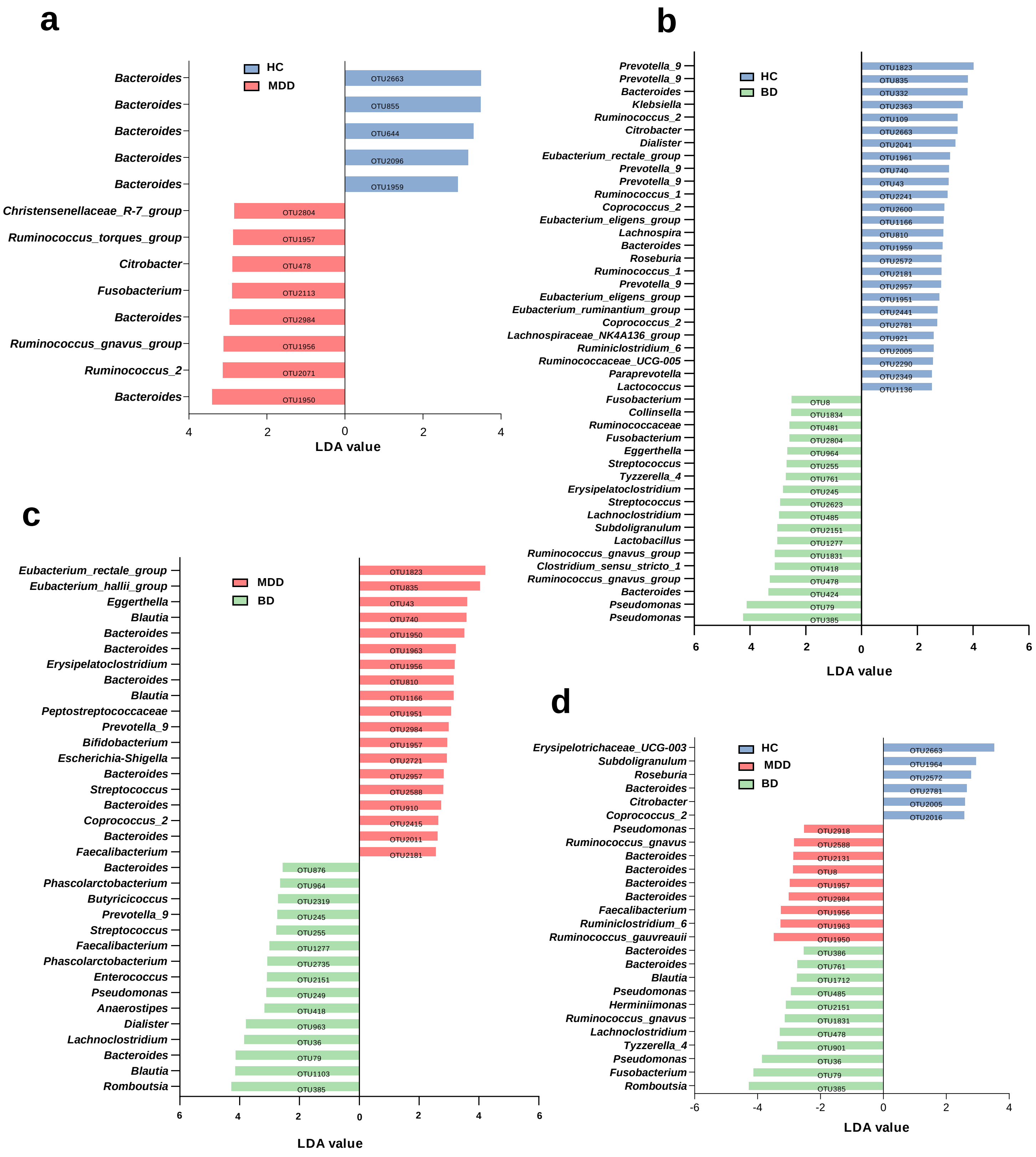

Figure. S4. Discriminative OTUs observed in the pairwise comparisons between the three groups. Using LEfSe analysis, the OTUs responsible for the discrimination among three groups were identified (LDA >2.5, fold change >2).

Figure. S5

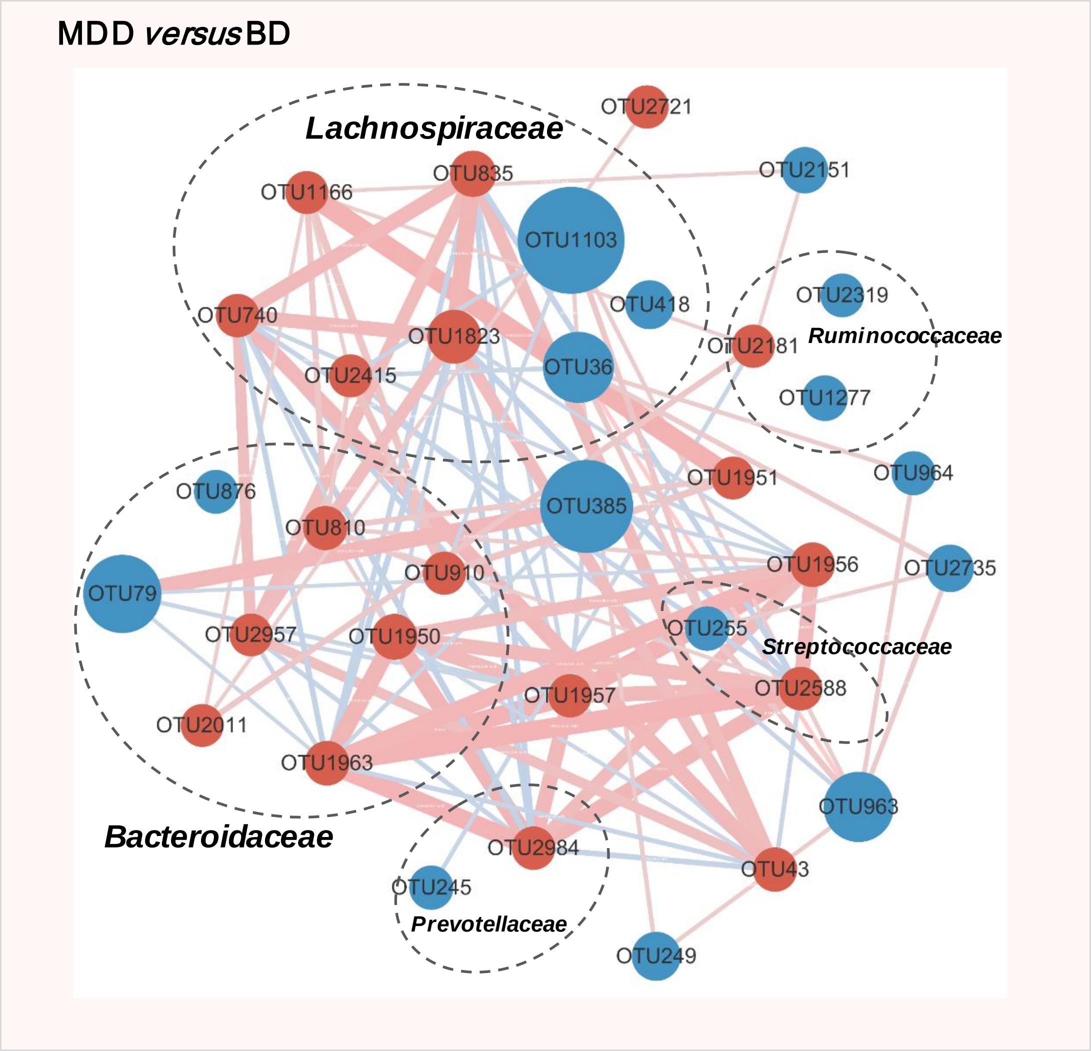

Figure. S5. Co-occurrence networks deduced from the relative abundance of differential OTUs between MDD and BD. The differential OTUs between MDD and BD groups were identified by LDA (LDA>2.5 and fold change>2). Totally, 34 OTUs were responsible for this discrimination. Compared with BD group, the MDD group was mainly characterized by altered covarying OTUs belonging to Bacteroidaceae and Lachnospiraceae. Red dots, increased relative abundance in MDD; blue dots, increased relative abundance in BD. OTUs annotated to family level were marked. Edges between nodes indicate Spearman's correlation < - 0.35 (light blue), or >0.35 (light red), edges thickness indicate p value (p<0.05)..

Figure. S6

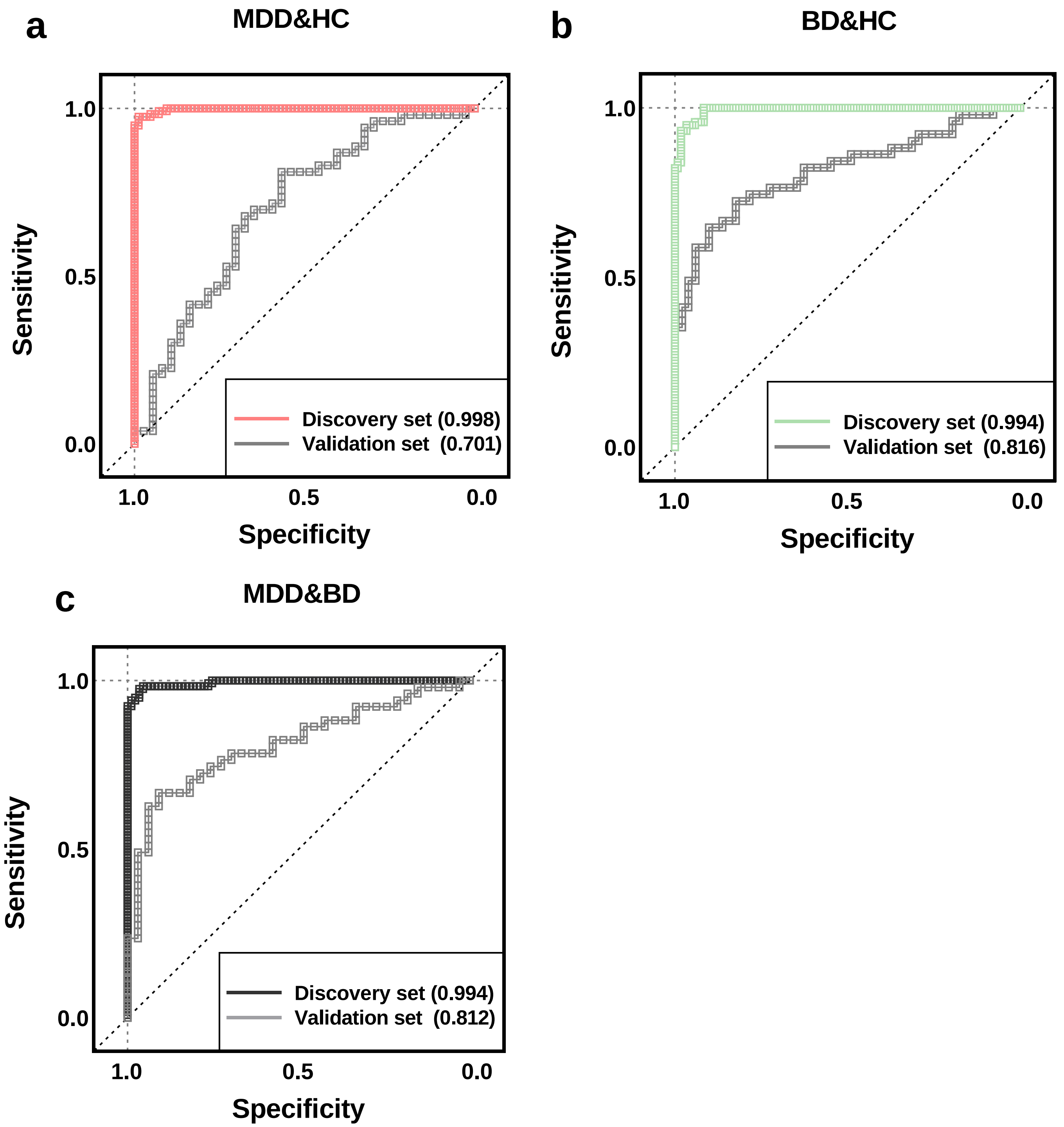

Figure. S6. Validation of diagnostic performance of microbial markers in discovery set. (a-c) Random Forest analysis was used to quantify the diagnostic performance in discovery set. The samples from discovery set were randomly divided into two groups. The group 1 was composed of 80% of samples from discovery set; the remaining samples were assigned to group 2. In group 1, this microbial panel enabled distinguishing the patients with MDD from those with BD or HCs, yielding a high diagnostic accuracy (MDD versus HC, AUC=0.998; BD versus HC, AUC=0.994; MDD versus BD, AUC=0.994). The diagnostic efficiency of this microbial classifier was further tested in group 2. Consequently, the diagnostic performance was confirmed with AUC values of at least 0.7 (MDD versus HC, AUC=0.701; BD versus HC, AUC=0.816; MDD versus BD, AUC=0.812; Group 1: HC, n=122; MDD, n=84; BD, n=118; Group 2: HC, n=49; MDD, n=38;BD, n=51.)

Supplementary Tables

Table S1. Detailed clinical characteristics of the subjects.

Table S2. Discriminatory microbes of family level among MDD, BD and HC groups.

Table S3. Discriminatory operational taxonomic units (OTUs) between MDD and HC groups.

Table S4. Discriminatory operational taxonomic units (OTUs) between BD and HC groups.

Table S5. Discriminatory operational taxonomic units (OTUs) between MDD and BD groups.

Table S6. Discriminatory operational taxonomic units (OTUs) among HC, MDD and BD groups.

[Tables below]

Table S1. Detailed clinical characteristics of the subjects.

Variables	Discovery set				Validation set			
	HC	MDD	BD	P	HC	MDD	BD	P
**Samples**	171	122	169	-	46	43	48	-
**Gender (male%)**	41.52%	36.89%	50.30%	0.06	52.17%	32.56%	64.58%	0.015
**Age**	26.85±5.48	26.54±4.07	25.59±8.41	0.177	45.4±7.1	37.13±9.15	21.87±7.44	<0.001
**BMI**	22.07±3.38	22.41±3.63	21.77±3.68	0.316	24.07±2.5	22.08±3.1	25.06±3.51	<0.001
**Area (Beijing/Zhejiang)**	148/23	122/0	19/150	-	24/22	43/0	28/20	-
**HAMD**	-	22.65±5.50	26.13±9.79	0.0005	-	23.47±4.64	28.49±10.34	0.001
**YMRS**	-	-	3.24±4.43	-	-	-	6.00±6.03	-
**Medication**	-	-	-	-	-	18/43	25/48	-

One-way ANOVA was used to compare the continuous variables including age, body mass index and clinical scales; Categorical variable (gender) was analyzed by Chi-square test.

Abbreviations: HC, Healthy controls; MDD, major depressive disorder; BD, bipolar disorder; BMI, Body Mass Index; HAMD, Hamilton Depression Scale; YMRS, Young Mania Rating Scale.

Table S2. Discriminatory microbes of family level among MDD, BD and HC groups.

Family level	HC		MDD		BD		p-value (Kruskal-Wallis test)			
	mean	sd	mean	sd	mean	sd	BD/HC	MDD/BD	MDD/HC	3 groups
Bacteroidaceae	1.4E-01	1.8E-01	1.6E-01	1.7E-01	1.0E-01	1.3E-01	0.199	0.002	0.048	0.07
Enterobacteriaceae	6.7E-02	1.5E-01	4.3E-02	1.1E-01	6.8E-02	1.2E-01	0.021	0.012	0.832	0.25
Bifidobacteriaceae	5.2E-02	8.3E-02	6.7E-02	8.4E-02	6.0E-02	8.4E-02	0.174	0.004	0.095	0.14
Veillonellaceae	3.4E-02	8.4E-02	4.5E-02	1.0E-01	3.0E-02	9.4E-02	0.068	0.005	0.251	0.16
Pseudomonadaceae	4.4E-04	8.3E-04	6.7E-04	4.2E-03	7.1E-02	1.8E-01	0.04	<0.001	<0.001	<0.001
Streptococcaceae	1.5E-02	3.9E-02	9.2E-03	1.5E-02	1.9E-02	3.6E-02	0.835	0.001	0.005	0.002
Peptostreptococcaceae	1.4E-02	2.8E-02	1.2E-02	2.9E-02	1.7E-02	2.3E-02	0.029	<0.001	0.003	<0.001
Acidaminococcaceae	1.0E-02	2.1E-02	1.6E-02	2.8E-02	1.2E-02	3.0E-02	0.518	0.003	0.023	0.003
Alcaligenaceae	5.1E-03	1.0E-02	5.3E-03	7.4E-03	3.4E-03	9.6E-03	0.002	<0.001	0.059	<0.001
Clostridiaceae_1	2.4E-03	5.3E-03	2.7E-03	6.6E-03	6.2E-03	1.5E-02	0.422	0.001	0.005	0.001
Lactobacillaceae	2.5E-03	1.1E-02	1.2E-03	3.9E-03	5.5E-03	2.4E-02	0.849	0.001	0.001	<0.001
Fusobacteriaceae	6.4E-04	3.9E-03	3.4E-03	1.4E-02	2.5E-03	8.3E-03	0.733	<0.001	<0.001	<0.001
Bacteroidales_S24-7_group	1.6E-03	8.0E-03	1.0E-03	4.8E-03	2.5E-03	1.2E-02	0.313	<0.001	<0.001	<0.001
Enterococcaceae	2.4E-03	2.4E-02	4.7E-04	2.3E-03	1.5E-03	9.8E-03	0.162	<0.001	0.017	0.001
norank_o__Mollicutes_RF9	1.1E-03	3.2E-03	1.1E-03	3.9E-03	1.1E-03	6.0E-03	0.264	0.003	0.101	0.01
Family_XIII	7.5E-04	9.1E-04	7.9E-04	1.5E-03	1.3E-03	1.9E-03	0.148	<0.001	0.016	0.001
Micrococcaceae	9.7E-05	4.0E-04	6.0E-05	1.7E-04	1.6E-03	1.5E-02	0.105	<0.001	0.002	<0.001
unclassified_o__Clostridiales	7.2E-04	9.4E-04	6.0E-04	8.1E-04	4.0E-04	1.1E-03	<0.001	<0.001	0.093	<0.001

norank_p__Saccharibacteria	4.0E-04	1.0E-03	2.4E-04	4.1E-04	6.4E-04	1.6E-03	0.159	<0.001	<0.001	<0.001
Leuconostocaceae	4.9E-04	2.0E-03	6.0E-04	2.2E-03	2.5E-04	8.4E-04	0.702	0.017	0.028	0.027
Actinomycetaceae	2.8E-04	4.9E-04	2.9E-04	5.1E-04	6.2E-04	1.0E-03	0.574	<0.001	<0.001	<0.001
Comamonadaceae	2.0E-05	6.9E-05	9.5E-06	4.0E-05	7.3E-04	7.1E-03	0.076	<0.001	<0.001	<0.001
unclassified_p__Firmicutes	2.9E-04	4.4E-04	3.0E-04	3.0E-04	2.2E-04	3.1E-04	0.002	<0.001	0.128	<0.001
Oxalobacteraceae	3.9E-05	1.0E-04	3.5E-05	9.6E-05	5.9E-04	1.5E-03	0.102	<0.001	<0.001	<0.001
Carnobacteriaceae	1.5E-04	2.9E-04	1.0E-04	2.0E-04	3.8E-04	9.6E-04	0.309	<0.001	0.001	<0.001
unclassified_k__norank	2.2E-04	3.7E-04	2.5E-04	2.6E-04	1.8E-04	3.3E-04	<0.001	<0.001	0.153	<0.001
Family_XI_o__Clostridiales	8.9E-05	2.0E-04	1.1E-04	3.6E-04	3.4E-04	1.1E-03	0.797	0.014	0.016	0.018
unclassified_o__Bacteroidales	2.0E-04	4.4E-04	2.3E-04	6.2E-04	1.0E-04	3.1E-04	0.001	<0.001	0.469	<0.001
norank_c__Cyanobacteria	1.3E-04	4.5E-04	2.0E-05	5.6E-05	2.1E-04	1.0E-03	0.007	<0.001	0.252	0.001
Eubacteriaceae	1.5E-05	5.1E-05	1.8E-04	1.6E-03	1.3E-04	3.4E-04	0.002	<0.001	0.002	<0.001
unclassified_c__Gammaproteobacteria	1.2E-04	2.2E-04	8.4E-05	1.6E-04	8.1E-05	1.8E-04	0.075	<0.001	0.008	<0.001
Family_XI_o__Bacillales	7.2E-05	1.7E-04	5.5E-05	1.2E-04	1.2E-04	2.7E-04	0.491	0.003	0.014	0.006
Aerococcaceae	1.2E-05	4.3E-05	1.4E-05	5.6E-05	1.9E-04	1.4E-03	0.725	<0.001	<0.001	<0.001
Synergistaceae	3.7E-05	2.0E-04	2.6E-05	7.8E-05	1.3E-04	4.0E-04	0.984	0.01	0.019	0.015
Burkholderiaceae	2.8E-05	8.6E-05	1.7E-05	5.0E-05	6.9E-05	1.9E-04	0.674	<0.001	<0.001	<0.001
Corynebacteriaceae	1.4E-05	2.8E-05	1.2E-05	2.7E-05	8.4E-05	3.0E-04	0.579	<0.001	<0.001	<0.001
Staphylococcaceae	8.4E-06	5.0E-05	8.6E-06	2.6E-05	7.9E-05	7.9E-04	0.652	<0.001	0.001	<0.001

Table S3. Discriminatory operational taxonomic units (OTUs) between MDD and HC groups.

OTU ID	Taxonomic Assignment			Relative Abundance				LDA value	LDA p-value	Enrichiment
	Phyla	Family	Genus	HC(mean)	HC(SD)	MDD(mean)	MDD(SD)			
OTU1950	Bacteroidetes	Bacteroidaceae	Bacteroides	3.9E-03	1.1E-02	8.5E-03	1.8E-02	3.41	0.003	MDD
OTU1956	Bacteroidetes	Bacteroidaceae	Bacteroides	2.4E-03	7.1E-03	5.1E-03	1.0E-02	3.11	0.003	MDD
OTU1957	Bacteroidetes	Bacteroidaceae	Bacteroides	1.0E-03	3.0E-03	2.6E-03	5.4E-03	2.86	<0.001	MDD
OTU1959	Bacteroidetes	Bacteroidaceae	Bacteroides	2.1E-03	8.1E-03	5.9E-04	2.2E-03	2.89	0.032	HC
OTU2071	Bacteroidetes	Bacteroidaceae	Bacteroides	2.5E-03	5.6E-03	5.3E-03	1.1E-02	3.13	0.002	MDD
OTU2096	Firmicutes	Christensenellaceae	Christensenellaceae_R-7	4.3E-03	1.5E-02	1.4E-03	4.4E-03	3.16	0.039	HC
OTU2113	Firmicutes	Lachnospiraceae	Ruminococcus_torques	8.8E-04	1.2E-03	2.3E-03	5.0E-03	2.89	0.002	MDD
OTU2663	Proteobacteria	Enterobacteriaceae	Citrobacter	8.2E-03	4.6E-02	9.9E-04	4.0E-03	3.48	<0.001	HC
OTU2804	Fusobacteria	Fusobacteriaceae	Fusobacterium	8.0E-05	6.3E-04	1.4E-03	9.3E-03	2.84	0.032	MDD
OTU2984	Bacteroidetes	Bacteroidaceae	Bacteroides	1.1E-03	2.6E-03	2.8E-03	5.2E-03	2.95	0.001	MDD
OTU478	Firmicutes	Lachnospiraceae	Ruminococcus_gnavus	1.4E-03	5.0E-03	3.0E-03	8.5E-03	2.88	0.008	MDD
OTU644	Firmicutes	Ruminococcaceae	Ruminococcus_2	5.4E-03	2.0E-02	2.4E-03	1.0E-02	3.29	<0.001	HC
OTU855	Bacteroidetes	Bacteroidaceae	Bacteroides	1.1E-02	3.4E-02	5.6E-03	2.0E-02	3.48	0.04	HC

Table S4. Discriminatory operational taxonomic units (OTUs) between BD and HC groups.

OTU ID	Taxonomic Assignment			Relative Abundance				LDA value	LDA p-value	Enrichiment
	Phyla	Family	Genus	HC(mean)	HC(SD)	BD(mean)	BD(SD)			
OTU109	Firmicutes	Ruminococcaceae	Ruminococcus_2	9.7E-03	2.0E-02	4.2E-03	8.7E-03	3.45	0.001	HC
OTU1136	Firmicutes	Streptococcaceae	Lactococcus	7.4E-04	4.7E-03	2.5E-04	2.6E-03	2.52	0.001	HC
OTU1166	Firmicutes	Lachnospiraceae	Eubacterium_eligens	2.1E-03	5.6E-03	4.9E-04	1.7E-03	2.95	<0.001	HC
OTU1277	Firmicutes	Lactobacillaceae	Lactobacillus	3.4E-04	3.2E-03	2.7E-03	2.0E-02	3.02	0.001	BD
OTU1823	Bacteroidetes	Prevotellaceae	Prevotella_9	3.1E-02	9.8E-02	9.3E-03	4.9E-02	4.02	<0.001	HC
OTU1831	Firmicutes	Lachnospiraceae	Ruminococcus_gnavus	1.7E-03	5.4E-03	4.3E-03	1.1E-02	3.11	<0.001	BD
OTU1834	Actinobacteria	Coriobacteriaceae	Collinsella	2.5E-04	9.4E-04	8.5E-04	6.2E-03	2.52	0.012	BD
OTU1951	Firmicutes	Lachnospiraceae	Eubacterium_eligens	1.6E-03	5.7E-03	4.5E-04	1.5E-03	2.79	<0.001	HC
OTU1959	Bacteroidetes	Bacteroidaceae	Bacteroides	2.1E-03	8.1E-03	2.7E-04	1.2E-03	2.91	0.002	HC
OTU1961	Firmicutes	Lachnospiraceae	Eubacterium_rectale	4.6E-03	1.3E-02	1.9E-03	3.3E-03	3.17	<0.001	HC
OTU2005	Firmicutes	Ruminococcaceae	Ruminiclostridium_6	7.9E-04	3.8E-03	1.0E-04	6.2E-04	2.59	<0.001	HC
OTU2041	Firmicutes	Veillonellaceae	Dialister	9.3E-03	4.3E-02	3.9E-03	1.2E-02	3.36	0.019	HC
OTU2151	Firmicutes	Ruminococcaceae	Subdoligranulum	1.3E-03	3.3E-03	3.2E-03	1.3E-02	3.02	<0.001	BD
OTU2181	Firmicutes	Ruminococcaceae	Ruminococcus_1	2.1E-03	5.2E-03	6.9E-04	1.9E-03	2.87	<0.001	HC
OTU2241	Firmicutes	Ruminococcaceae	Ruminococcus_1	3.9E-03	1.2E-02	1.7E-03	5.3E-03	3.09	0.013	HC
OTU2290	Firmicutes	Ruminococcaceae	Ruminococcaceae_UCG-005	1.1E-03	2.9E-03	3.6E-04	1.4E-03	2.56	0.005	HC
OTU2349	Bacteroidetes	Prevotellaceae	Paraprevotella	1.0E-03	5.0E-03	1.9E-04	8.7E-04	2.52	0.002	HC
OTU2363	Proteobacteria	Enterobacteriaceae	Klebsiella	2.0E-02	6.9E-02	9.4E-03	3.7E-02	3.63	<0.001	HC
OTU2441	Firmicutes	Lachnospiraceae	Eubacterium_ruminantium	1.6E-03	5.6E-03	6.0E-04	3.2E-03	2.73	0.002	HC
OTU245	Firmicutes	Erysipelotrichaceae	Erysipelatoclostridium	2.3E-04	1.1E-03	1.5E-03	6.0E-03	2.82	<0.001	BD

OTU255	Firmicutes	Streptococcaceae	Streptococcus	8.0E-04	1.7E-03	1.7E-03	3.9E-03	2.69	0.018	BD
OTU2572	Firmicutes	Lachnospiraceae	Roseburia	1.8E-03	6.4E-03	4.6E-04	1.1E-03	2.87	<0.001	HC
OTU2600	Firmicutes	Lachnospiraceae	Coprococcus_2	3.0E-03	8.9E-03	1.0E-03	5.3E-03	2.97	<0.001	HC
OTU2623	Firmicutes	Streptococcaceae	Streptococcus	5.6E-04	3.5E-03	2.2E-03	1.9E-02	2.92	<0.001	BD
OTU2663	Proteobacteria	Enterobacteriaceae	Citrobacter	8.2E-03	4.6E-02	1.1E-03	5.2E-03	3.44	0.002	HC
OTU2781	Firmicutes	Lachnospiraceae	Coprococcus_2	1.5E-03	4.1E-03	6.3E-04	2.6E-03	2.72	<0.001	HC
OTU2804	Fusobacteria	Fusobacteriaceae	Fusobacterium	8.0E-05	6.3E-04	9.2E-04	5.0E-03	2.59	0.01	BD
OTU2957	Bacteroidetes	Prevotellaceae	Prevotella_9	1.8E-03	9.4E-03	3.0E-04	2.9E-03	2.85	<0.001	HC
OTU332	Bacteroidetes	Bacteroidaceae	Bacteroides	2.5E-02	6.0E-02	1.1E-02	3.3E-02	3.81	0.015	HC
OTU385	Proteobacteria	Pseudomonadaceae	Pseudomonas	2.2E-04	4.3E-04	4.1E-02	1.1E-01	4.25	<0.001	BD
OTU418	Firmicutes	Clostridiaceae_1	Clostridium_sensu_stricto_1	2.2E-03	5.2E-03	5.4E-03	1.4E-02	3.12	0.027	BD
OTU424	Bacteroidetes	Bacteroidaceae	Bacteroides	1.2E-03	3.5E-03	6.5E-03	2.5E-02	3.34	0.004	BD
OTU43	Bacteroidetes	Prevotellaceae	Prevotella_9	5.2E-03	2.2E-02	1.6E-03	9.6E-03	3.12	0.004	HC
OTU478	Firmicutes	Lachnospiraceae	Ruminococcus_gnavus	1.4E-03	5.0E-03	5.3E-03	1.3E-02	3.28	<0.001	BD
OTU481	Firmicutes	Ruminococcaceae	norank_Ruminococcaceae	4.4E-04	8.0E-04	1.2E-03	2.8E-03	2.58	0.017	BD
OTU485	Firmicutes	Lachnospiraceae	Lachnoclostridium	4.4E-04	1.4E-03	2.2E-03	6.2E-03	2.95	<0.001	BD
OTU740	Bacteroidetes	Prevotellaceae	Prevotella_9	3.9E-03	1.8E-02	8.7E-04	5.9E-03	3.13	0.008	HC
OTU761	Firmicutes	Lachnospiraceae	Tyzzerella_4	6.4E-04	2.3E-03	1.6E-03	4.5E-03	2.7	0.022	BD
OTU79	Proteobacteria	Pseudomonadaceae	Pseudomonas	2.1E-04	4.0E-04	2.9E-02	6.8E-02	4.12	<0.001	BD
OTU8	Fusobacteria	Fusobacteriaceae	Fusobacterium	2.3E-04	1.3E-03	1.0E-03	4.9E-03	2.51	<0.001	BD
OTU810	Firmicutes	Lachnospiraceae	Lachnospira	2.9E-03	8.5E-03	1.4E-03	5.6E-03	2.93	<0.001	HC
OTU835	Bacteroidetes	Prevotellaceae	Prevotella_9	1.7E-02	5.4E-02	3.2E-03	1.6E-02	3.82	<0.001	HC
OTU921	Firmicutes	Lachnospiraceae	Lachnospiraceae_NK4A136	1.4E-03	4.6E-03	6.1E-04	1.7E-03	2.59	<0.001	HC
OTU964	Actinobacteria	Coriobacteriaceae	Eggerthella	4.6E-04	1.2E-03	1.4E-03	4.7E-03	2.67	<0.001	BD

Table S5. Discriminatory operational taxonomic units (OTUs) between MDD and BD groups.

OTU ID	Taxonomic Assignment			Relative Abundance				LDA value	LDA p-value	Enrichiment group
	Phyla	Family	Genus	MDD(mean)	MDD(SD)	BD(mean)	BD(SD)			
OTU963	Firmicutes	Veillonellaceae	Dialister	1.1E-02	1.5E-02	2.3E-02	3.5E-02	3.8	0.001	BD
OTU255	Firmicutes	Streptococcaceae	Streptococcus	6.0E-04	1.0E-03	1.7E-03	3.9E-03	2.78	0.001	BD
OTU2588	Firmicutes	Streptococcaceae	Streptococcus	2.2E-03	3.6E-03	9.5E-04	2.8E-03	2.81	<0.001	MDD
OTU1277	Firmicutes	Ruminococcaceae	Faecalibacterium	3.6E-04	2.7E-03	2.7E-03	2.0E-02	3.01	0.047	BD
OTU2181	Firmicutes	Ruminococcaceae	Faecalibacterium	1.4E-03	4.0E-03	6.9E-04	1.9E-03	2.56	0.022	MDD
OTU2319	Firmicutes	Ruminococcaceae	Butyricicoccus	3.9E-04	2.1E-03	1.1E-03	7.7E-03	2.71	0.011	BD
OTU249	Proteobacteria	Pseudomonadaceae	Pseudomonas	2.6E-03	3.3E-03	5.4E-03	9.1E-03	3.12	0.001	BD
OTU245	Bacteroidetes	Prevotellaceae	Prevotella_9	3.2E-04	1.4E-03	1.5E-03	6.0E-03	2.74	<0.001	BD
OTU2984	Bacteroidetes	Prevotellaceae	Prevotella_9	2.8E-03	5.2E-03	8.3E-04	2.7E-03	2.99	<0.001	MDD
OTU1951	Firmicutes	Peptostreptococcaceae	unclassified	3.1E-03	1.1E-02	4.5E-04	1.5E-03	3.08	<0.001	MDD
OTU385	Firmicutes	Peptostreptococcaceae	Romboutsia	3.1E-04	1.9E-03	4.1E-02	1.1E-01	4.28	<0.001	BD
OTU1103	Firmicutes	Lachnospiraceae	Blautia	2.6E-02	6.9E-02	5.2E-02	1.0E-01	4.14	0.014	BD
OTU1166	Firmicutes	Lachnospiraceae	Blautia	3.8E-03	1.9E-02	4.9E-04	1.7E-03	3.15	<0.001	MDD
OTU1823	Firmicutes	Lachnospiraceae	Eubacterium_rectale_group	4.4E-02	1.1E-01	9.3E-03	4.9E-02	4.22	<0.001	MDD
OTU2415	Firmicutes	Lachnospiraceae	Coprococcus_2	1.4E-03	3.6E-03	5.4E-04	3.4E-03	2.64	<0.001	MDD
OTU36	Firmicutes	Lachnospiraceae	Lachnoclostridium	9.0E-03	1.1E-02	2.3E-02	3.3E-02	3.84	<0.001	BD
OTU418	Firmicutes	Lachnospiraceae	Anaerostipes	2.3E-03	5.9E-03	5.4E-03	1.4E-02	3.18	0.002	BD
OTU740	Firmicutes	Lachnospiraceae	Blautia	8.4E-03	2.6E-02	8.7E-04	5.9E-03	3.58	0.001	MDD
OTU835	Firmicutes	Lachnospiraceae	Eubacterium_hallii_group	2.5E-02	6.3E-02	3.2E-03	1.6E-02	4.04	<0.001	MDD

OTU1956	Firmicutes	Erysipelotrichaceae	Erysipelatoclostridium	5.1E-03	1.0E-02	1.8E-03	4.9E-03	3.19	<0.001	MDD
OTU2151	Firmicutes	Enterococcaceae	Enterococcus	7.7E-04	1.3E-03	3.2E-03	1.3E-02	3.09	0.031	BD
OTU2721	Proteobacteria	Enterobacteriaceae	Escherichia-Shigella	2.6E-03	1.6E-02	8.3E-04	5.0E-03	2.93	0.025	MDD
OTU43	Actinobacteria	Coriobacteriaceae	Eggerthella	1.0E-02	2.8E-02	1.6E-03	9.6E-03	3.61	0.003	MDD
OTU1957	Actinobacteria	Bifidobacteriaceae	Bifidobacterium	2.6E-03	5.4E-03	7.4E-04	2.3E-03	2.93	<0.001	MDD
OTU1950	Bacteroidetes	Bacteroidaceae	Bacteroides	8.5E-03	1.8E-02	2.8E-03	7.0E-03	3.51	<0.001	MDD
OTU1963	Bacteroidetes	Bacteroidaceae	Bacteroides	5.3E-03	7.3E-03	1.8E-03	4.0E-03	3.23	<0.001	MDD
OTU2011	Bacteroidetes	Bacteroidaceae	Bacteroides	1.1E-03	6.6E-03	4.1E-04	1.7E-03	2.62	0.001	MDD
OTU2957	Bacteroidetes	Bacteroidaceae	Bacteroides	1.4E-03	9.8E-03	3.0E-04	2.9E-03	2.82	0.001	MDD
OTU79	Bacteroidetes	Bacteroidaceae	Bacteroides	3.5E-04	2.4E-03	2.9E-02	6.8E-02	4.14	<0.001	BD
OTU810	Bacteroidetes	Bacteroidaceae	Bacteroides	3.9E-03	8.9E-03	1.4E-03	5.6E-03	3.15	0.001	MDD
OTU876	Bacteroidetes	Bacteroidaceae	Bacteroides	5.7E-04	2.3E-03	1.3E-03	3.1E-03	2.56	0.044	BD
OTU910	Bacteroidetes	Bacteroidaceae	Bacteroides	1.5E-03	3.4E-03	5.9E-04	1.5E-03	2.73	<0.001	MDD
OTU2735	Firmicutes	Acidaminococcaceae	Phascolarctobacterium	1.5E-03	3.6E-03	4.1E-03	1.5E-02	3.08	0.006	BD
OTU964	Firmicutes	Acidaminococcaceae	Phascolarctobacterium	5.1E-04	1.5E-03	1.4E-03	4.7E-03	2.65	<0.001	BD

Table S6. Discriminatory operational taxonomic units (OTUs) among HC, MDD and BD groups.

OTU ID	Taxonomic Assignment			Relative Abundance						LDA value	LDA p-value	Enrichiment
	Phyla	Family	Genus	HC(mean)	HC(SD)	MDD(mean)	MDD(SD)	BD(mean)	BD(SD)			
OTU1712	Proteobacteria	Pseudomonadaceae	Pseudomonas	1.4E-02	2.5E-02	9.0E-03	1.1E-02	2.3E-02	3.3E-02	2.75	0.001	BD
OTU1831	Firmicutes	Lachnospiraceae	Ruminococcus_gnavus	1.4E-03	5.0E-03	3.0E-03	8.5E-03	5.3E-03	1.3E-02	3.13	0.001	BD
OTU1950	Bacteroidetes	Bacteroidaceae	Bacteroides	1.7E-03	3.2E-03	1.1E-03	2.2E-03	9.5E-04	2.2E-03	3.48	<0.001	MDD
OTU1956	Bacteroidetes	Bacteroidaceae	Bacteroides	4.5E-03	5.7E-03	3.2E-03	3.1E-03	2.7E-03	4.2E-03	3.25	<0.001	MDD
OTU1957	Bacteroidetes	Bacteroidaceae	Bacteroides	1.0E-03	3.0E-03	2.6E-03	5.4E-03	7.4E-04	2.3E-03	2.98	<0.001	MDD
OTU1963	Bacteroidetes	Bacteroidaceae	Bacteroides	7.9E-04	3.8E-03	1.1E-04	4.5E-04	1.0E-04	6.2E-04	3.27	<0.001	MDD
OTU1964	Firmicutes	Ruminococcaceae	Faecalibacterium	1.3E-03	3.3E-03	7.7E-04	1.3E-03	3.2E-03	1.3E-02	2.95	<0.001	HC
OTU2005	Firmicutes	Ruminococcaceae	Ruminiclostridium_6	8.2E-03	4.6E-02	9.9E-04	4.0E-03	1.1E-03	5.2E-03	2.6	<0.001	HC
OTU2016	Firmicutes	Lachnospiraceae	Ruminococcus_gauvreauii	1.5E-03	4.1E-03	1.2E-03	5.7E-03	6.3E-04	2.6E-03	2.58	<0.001	HC
OTU2131	Firmicutes	Erysipelotrichaceae	Erysipelotrichaceae_UCG-003	3.9E-03	1.1E-02	8.5E-03	1.8E-02	2.8E-03	7.0E-03	2.86	0.001	MDD
OTU2151	Firmicutes	Ruminococcaceae	Subdoligranulum	2.8E-06	8.3E-06	4.5E-06	4.1E-05	5.3E-04	1.5E-03	3.1	<0.001	BD
OTU2572	Firmicutes	Lachnospiraceae	Roseburia	1.8E-03	6.4E-03	7.0E-04	1.4E-03	4.6E-04	1.1E-03	2.79	<0.001	HC
OTU2588	Bacteroidetes	Bacteroidaceae	Bacteroides	1.7E-03	5.4E-03	3.1E-03	7.8E-03	4.3E-03	1.1E-02	2.84	<0.001	MDD
OTU2663	Proteobacteria	Enterobacteriaceae	Citrobacter	5.4E-06	1.6E-05	9.8E-06	2.4E-05	2.4E-06	9.4E-06	3.53	<0.001	HC

OTU2781	Firmicutes	Lachnospiraceae	Coprococcus_2	1.7E-03	3.6E-03	2.2E-03	3.6E-03	9.5E-04	2.8E-03	2.66	<0.001	HC
OTU2918	Bacteroidetes	Bacteroidaceae	Bacteroides	4.3E-06	1.3E-05	2.8E-06	1.2E-05	8.1E-06	2.1E-05	2.52	<0.001	MDD
OTU2984	Bacteroidetes	Bacteroidaceae	Bacteroides	3.4E-03	6.2E-03	5.3E-03	7.3E-03	1.8E-03	4.0E-03	3.01	<0.001	MDD
OTU36	Firmicutes	Lachnospiraceae	Blautia	2.1E-04	4.0E-04	3.5E-04	2.4E-03	2.9E-02	6.8E-02	3.85	<0.001	BD
OTU385	Proteobacteria	Pseudomonadaceae	Pseudomonas	8.9E-03	2.0E-02	7.2E-03	2.0E-02	1.1E-02	1.7E-02	4.27	<0.001	BD
OTU386	Proteobacteria	Oxalobacteraceae	Herminiimonas	1.2E-03	3.1E-03	1.4E-03	2.8E-03	8.1E-04	2.6E-03	2.53	<0.001	BD
OTU478	Firmicutes	Lachnospiraceae	Ruminococcus_gnavus	4.4E-04	1.4E-03	1.2E-03	4.2E-03	2.2E-03	6.2E-03	3.29	<0.001	BD
OTU485	Firmicutes	Lachnospiraceae	Lachnoclostridium	2.2E-04	4.3E-04	3.1E-04	1.9E-03	4.1E-02	1.1E-01	2.94	<0.001	BD
OTU761	Firmicutes	Lachnospiraceae	Tyzzerella_4	1.1E-03	2.6E-03	2.8E-03	5.2E-03	8.3E-04	2.7E-03	2.74	<0.001	BD
OTU79	Proteobacteria	Pseudomonadaceae	Pseudomonas	2.3E-04	1.3E-03	1.9E-03	1.0E-02	1.0E-03	4.9E-03	4.13	<0.001	BD
OTU8	Fusobacteria	Fusobacteriaceae	Fusobacterium	2.4E-03	7.1E-03	5.1E-03	1.0E-02	1.8E-03	4.9E-03	2.88	<0.001	MDD
OTU901	Firmicutes	Peptostreptococcaceae	Romboutsia	6.4E-04	2.3E-03	1.1E-03	4.1E-03	1.6E-03	4.5E-03	3.37	<0.001	BD
